# Autophagy Improves ARA-Rich TAG Accumulation in *Mortierella alpina* by Regulating Resource Allocation

**DOI:** 10.1128/spectrum.01300-21

**Published:** 2022-02-09

**Authors:** Hengqian Lu, Haiqin Chen, Xin Tang, Qin Yang, Hao Zhang, Yong Q. Chen, Wei Chen

**Affiliations:** a State Key Laboratory of Food Science and Technology, Jiangnan University, Wuxi, Jiangsu, China; b School of Food Science and Technology, Jiangnan University, Wuxi, Jiangsu, China; c National Engineering Research Center for Functional Food, Jiangnan University, Wuxi, Jiangsu, China; d Yangzhou Institute of Food Biotechnology, Jiangnan University, Yangzhou, China; e Department of Cancer Biology, Wake Forest School of Medicine, Winston-Salem, North Carolina, USA; University of Molise

**Keywords:** autophagy, lipid accumulation, microbial oil, *Mortierella alpina*, nitrogen limitation, resource reallocation

## Abstract

The present study was designed to explore the possibility of improving lipid production in oleaginous filamentous fungus Mortierella alpina based on an autophagy regulation strategy. According to multiomics information, vacuolate-centered macroautophagy was identified as the main type of autophagy in M. alpina under nitrogen-limited conditions. Mutation of autophagy-related gene *MAatg8* led to impaired fatty acid synthesis, while overexpression of both *MAatg8* and phosphatidylserine decarboxylases (*MApsd2*) showed promoting effects on fatty acid synthesis. *MAatg8* overexpression strain with external supply of ethanolamine significantly increased arachidonic acid (ARA)-rich triacylglycerol (TAG) and biomass synthesis in *M. alpina*, and the final fatty acid content increased by approximately 110% compared with that in the wild-type strain. Metabolomics and lipidomics analyses revealed that cell autophagy enhanced the recycling of preformed carbon, nitrogen, and lipid in mycelium, and the released carbon skeleton and energy were contributed to the accumulation of TAG in *M. alpina*. This study suggests that regulation of autophagy-related MAatg8-phosphatidylethanolamine (MAatg8-PE) conjugation system could be a promising strategy for attaining higher lipid production and biomass growth. The mechanism of autophagy in regulating nitrogen limitation-induced lipid accumulation elucidated in this study provides a reference for development of autophagy-based strategies for improving nutrient use efficiency and high value-added lipid production by oleaginous microorganism.

**IMPORTANCE** Studies have indicated that functional oil accumulation occurs in oleaginous microorganisms under nitrogen limitation. However, until now, large-scale application of nitrogen-deficiency strategies was limited by low biomass. Therefore, the identification of the critical nodes of nitrogen deficiency-induced lipid accumulation is urgently needed to further guide functional oil production. The significance of our research is in uncovering the function of cell autophagy in the ARA-rich TAG accumulation of oleaginous fungus *M. alpina* and demonstrating the feasibility of improving lipid production based on an autophagy regulation strategy at the molecular and omics levels. Our study proves that regulation of cell autophagy through the MAatg8-PE conjugation system-related gene overexpression or exogenous supply of ethanolamine would be an efficient strategy to increase and maintain biomass productivity when high TAG content is obtained under nitrogen deficiency, which could be useful for the development of new strategies that will achieve more biomass and maximal lipid productivity.

## INTRODUCTION

Exhaustion and pollution of traditional fuel supplies have become increasingly serious problems with economic development and population growth. Therefore, environmentally sustainable and ecofriendly substitutes are needed. Microbial oil has been the focus of intense research in recent years and is thought to be an environmentally friendly alternative energy source that can be produced sustainably (1–3). Studies have indicated that triacylglycerol (TAG) hyperaccumulation occurs in oleaginous microorganisms under nitrogen limitation (4–6). However, large-scale application of nitrogen-deficiency strategies is limited by low biomass. Therefore, the identification of the critical nodes of nitrogen limitation-induced lipid accumulation is urgently needed to further guide biodiesel and functional oil production.

Autophagy is highly conserved in eukaryotic cells and recognized to be a conjugation pathway that attaches autophagy-related gene 8 (*atg8*) to phosphatidylethanolamine (PE), which then coats emerging autophagic membranes and assists with cargo recruitment, vesicle enclosure, and subsequent vesicle docking with the tonoplast (7). Following nutrient stress, cell autophagy as a primary catabolic pathway is induced to deliver superfluous or damaged cytoplasmic material and organelles to the lysosomal/vacuole for degradation and recycling for cellular survival. In the past years, the relationship between autophagy and lipid metabolism has been demonstrated in various species (8–10). Originally, cell autophagy was believed to be required for the breakdown of lipid droplets (LDs) in hepatocytes and mouse livers (9). In recent years, contrary to the role of autophagy in hepatocytes, autophagy has been shown to have the function of regulating the formation of LDs in adipocytes, *Arabidopsis*, and fungi. (8, 10–13). Therefore, autophagy is a plausible target for efficient lipid production.

Oleaginous microorganisms are defined as the microbes which can accumulate lipids more than 20% of their dry cell weight (1, 14). The mechanisms of nitrogen limitation induction of lipid accumulation have been widely investigated; however, they remain ambiguous (4–6, 15). To date, a popular accepted view is that nitrogen limitation induces resource reallocation and metabolic reprogramming, which play a critical role in promoting the supply of precursors required for TAG synthesis (16). Specifically, under nitrogen limitation, biosynthesis pathways are downregulated while nutrient recycling and protein and amino acid degradation pathways are upregulated. This global metabolic alternation ensures cellular survival and energy homeostasis in response to nutrient stress. At the same time, the products from these degradation processes provide the carbon skeleton and energy required for fatty acid synthesis (6). Mortierella alpina strains are oleaginous model strains that can accumulate more than 50% lipids under nitrogen-limiting conditions (17, 18). Our previous multiomics analysis indicated that cell autophagy was significantly upregulated in M. alpina during the nitrogen stress-induced TAG accumulation process (19). However, it remains to be established whether autophagy affects neutral lipid accumulation during nutrient deprivation.

In this study, the MAatg8-PE conjugation system-related genes were overexpressed and knocked down in *M. alpina* to determine the effects of autophagy on cell growth and metabolism and to elucidate the underlying metabolic mechanisms of autophagy on nitrogen limitation-induced TAG accumulation. This study identifies a critical function for autophagy in lipid metabolism in *M. alpina* and reveals specific aspects of fungal metabolism that are controlled by this nutrient recycling system, which provides references for the construction of industrial strains and fermentation control for the efficient production of functional lipids.

## RESULTS

### Identification of genes involved in cell autophagy in *M. alpina* based on omics information.

GO enrichment analysis using the genome-wide annotated genes indicated that 166 autophagy-related genes were identified, which were involved in 17 autophagy-related biological processes (supplemental file 1). We further analyzed the fold change of the 166 genes prior to and after nitrogen limitation using our previously reported transcriptomics and proteomics data sets (19, 20). Among the 166 genes, 74 genes were identified at both mRNA and protein levels. We analyzed the correlation of expression of the 74 genes at the transcriptional and protein levels (Fig. S1, supplemental file 3) and found that most significantly changed genes (log 2 fold change > 1) were distributed in the first (up-up) and third (down-down) quartiles, indicating that the significantly changed genes were correlated at the transcriptional and protein levels.

**FIG 1:**
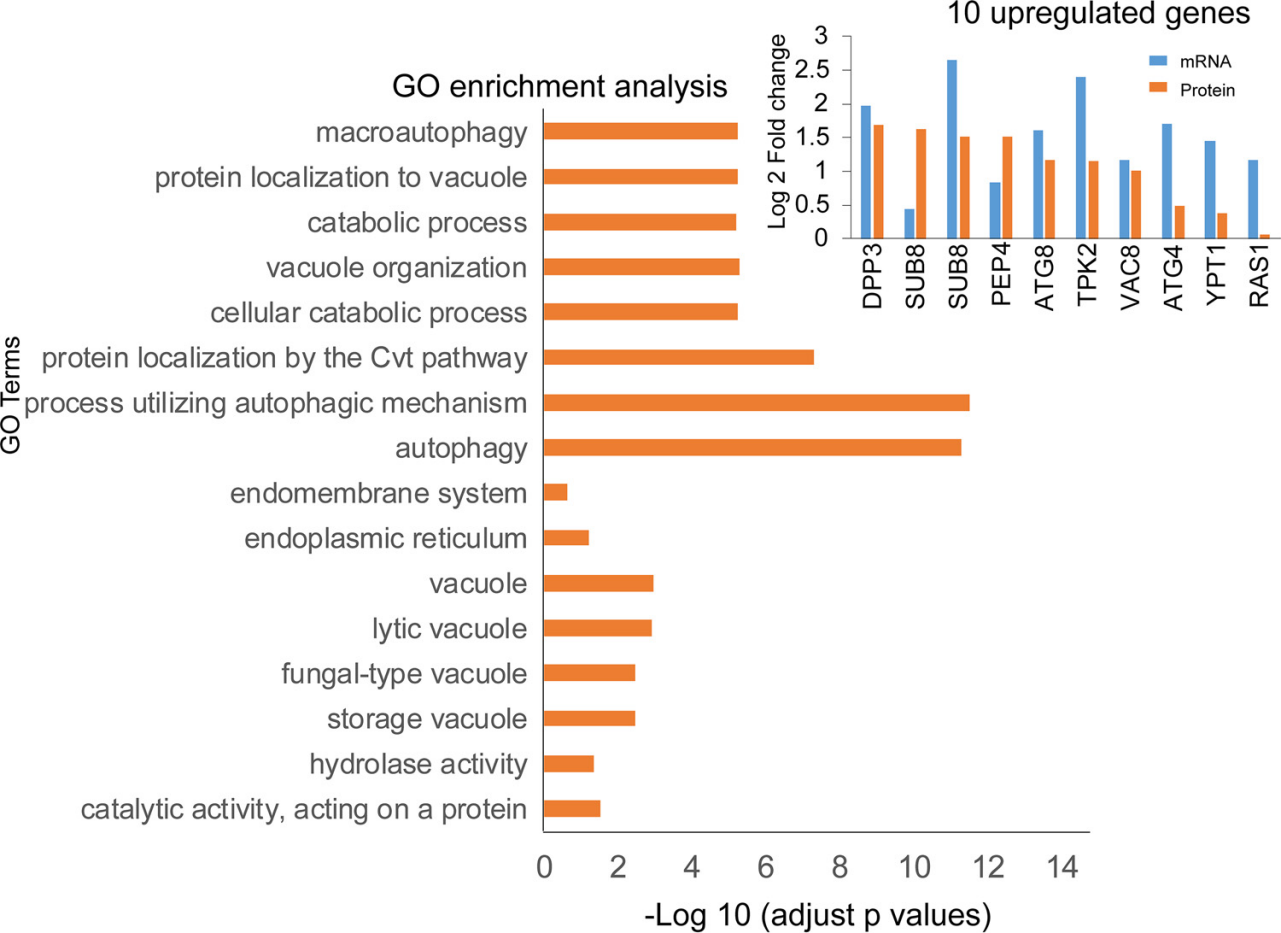
GO enrichment results of 10 upregulated genes involved in cell autophagy. Based on our previous transcriptomics and proteomics data sets, cell autophagy-related genes and proteins were selected in this study. The changes to these selected genes/proteins prior to and after nitrogen limitation were analyzed, and 10 autophagy-related genes and proteins were significantly upregulated prior to and after nitrogen limitation.

GO enrichment analysis showed that the upregulated genes mainly perform the catalytic activity function, acting on a protein associated with vacuoles or endoplasmic reticulum, and/or mainly participate in cellular catabolic process, protein localization by the Cvt pathway, protein localization to vacuole, and macroautophagy (Fig. 1). Therefore, macroautophagy centered on vacuoles was identified as the main type of autophagy that occurs during TAG biosynthesis in *M. alpina* induced by nitrogen limitation.

### The MAatg8-PE conjugation system is needed for the synthesis of total fatty acids under nitrogen-limiting conditions.

**FIG 2:**
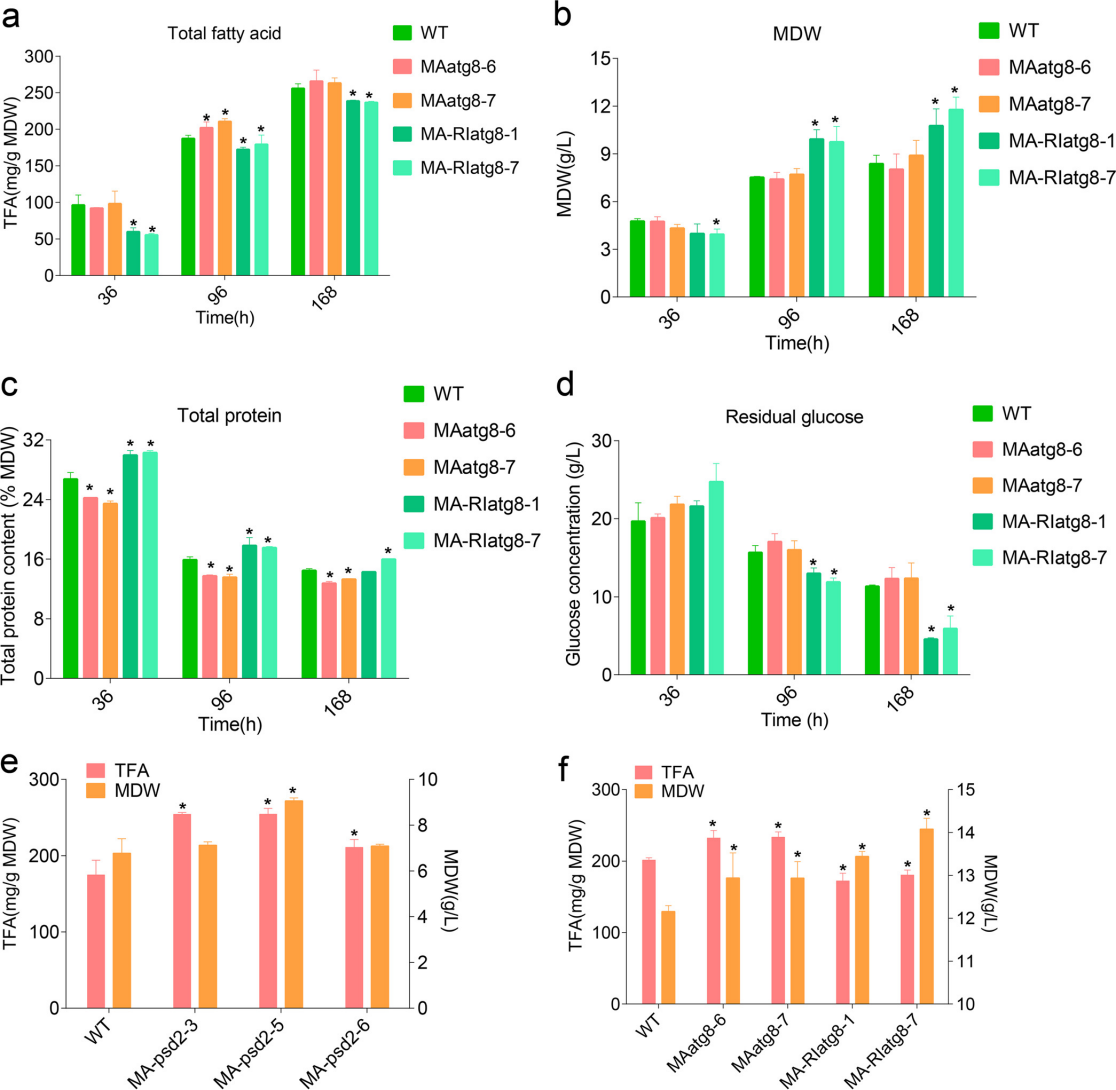
A MAatg8-PE conjugation system is needed for the synthesis of total fatty acids under nitrogen-limiting conditions. The time course of total fatty acids (a), mycelial dry weight (b), total protein (c), and residual glucose (d) in WT and *MAatg8* overexpression (*MAatg8-6*/*MA-atg8-7*) and interference (*MA-RIatg8-1*/*MA-RIatg8-7*) strains. (e) Total fatty acids and biomass in *MApsd2* overexpression strains (*MApsd2*-3/5/6) at 96 h. (f) Total fatty acids and biomass in WT and *MAatg8* overexpression and interference strains with ethanolamine treatment. *, *P < *0.05.

Based on the GO annotation and enrichment results, the autophagy-related gene *MAatg8*, which was found to be involved in various cell autophagy processes (supplemental file 1), was successfully overexpressed and knocked down in *M. alpina* (Fig. S2 to S4, supplemental file 3). As shown in Fig. 2a, compared with that in the wild-type (WT) strain, *MAatg8* knockdown significantly decreased the total fatty acid (TFA) level in *M. alpina* throughout the fermentation process, especially at 36 h, when the TFA levels decreased by 37.9 to 42.4%. Overexpression of *MAatg8* significantly increased the TFA content at 96 h (increased by 8.0 to 12.5%); however, *MAatg8* overexpression had no obvious effects on the TFA content at 36 and 168 h. The biomass level was significantly increased at 96 and 168 h in *MAatg8* knockdown strains (Fig. 2b), while overexpression of *MAatg8* had no effects on biomass. Compared with that in WT, the intracellular total protein content in *MAatg8* overexpression and knockdown strains was significantly decreased and increased (*P < *0.05), respectively (Fig. 2c). The glucose concentration in *MAatg8* interference strains was significantly lower (*P < *0.05) than that in WT and overexpression strains (Fig. 2d), indicating that knockdown of *MAatg8* significantly accelerated the consumption of glucose for biomass synthesis.

Autophagy is a conjugation pathway that attaches *atg8* to PE. It has been reported that phosphatidylserine decarboxylases (*psd2*) play an important role in PE synthesis and autophagosome formation (21). We further overexpressed *MApsd2* in *M. alpina* and found that *MApsd2* overexpression significantly increased the TFA level in *M. alpina* (Fig. 2e). Therefore, PE as the key component of the MAatg8-PE conjugation system may play an important role in TFA accumulation under nitrogen limitation. Rockenfeller et al. indicated that external supply of ethanolamine enhances the endogenous PE pool and positively regulates autophagy (22). Our results found that, compared with normal fermentation conditions (Fig. 2a and b), ethanolamine treatment (50 mM) significantly increased the biomass of the WT (61.96%), *MAatg8* overexpression (67.92 to 74.86%), and knockdown strains (35.48 to 44.41%) (Fig. 2f, Table S2, supplemental file 3) and enhanced a 7.59% and 10.82 to 14.87% increase of intracellular TFA content in WT and *MAatg8* overexpression strain, respectively, while ethanolamine treatment had no effects on the intracellular TFA content of *MAatg8* interference strains (increased by only 0.01 to 0.68%). The intracellular TFA content in *MAatg8* overexpression strains reached 233 mg/g mycelial dry weight (MDW) at 96 h with ethanolamine treatment and had a 15 to 16% increase compared with that in WT; however, there was only a 7 to 12% increase in nonethanolamine supplementary conditions (Fig. 2a and f, Table S2, supplemental file 3). Finally, the overexpression of *MAatg8* combined with the external supply of ethanolamine significantly increased the TFA content (approximately 3,000 mg/L) accumulation in *M. alpina* under nitrogen-limiting conditions, which was 110% higher than that in WT strains (Table S3, supplemental file 3).

Together, these results support the notion that autophagy positively regulates lipid accumulation in *M. alpina*. Intracellular PE levels may affect the lipid synthesis through the regulation of autophagy. PE is most likely to be a limiting factor for autophagy-mediated lipid accumulation in *MAatg8* overexpression strains.

### *MAatg8* regulates the remodeling of carbon and nitrogen metabolism in *M. alpina*.

To explore the underlying mechanisms of how *MAatg8* affects lipid biosynthesis in *M. alpina*, we compared the intracellular metabolites between WT, *MAatg8*, and *MARIatg8* strains at 36, 96, and 168 h (supplemental file 2). Principal-component analysis (PCA) showed that fermentation time had discernible effects on WT and *MAatg8* overexpression and knockdown strains. Metabolic profiles at 96 and 168 h were similar to each other but significantly different from that of the 36 h sample (Fig. S5a, supplemental file 3).

**FIG 3:**
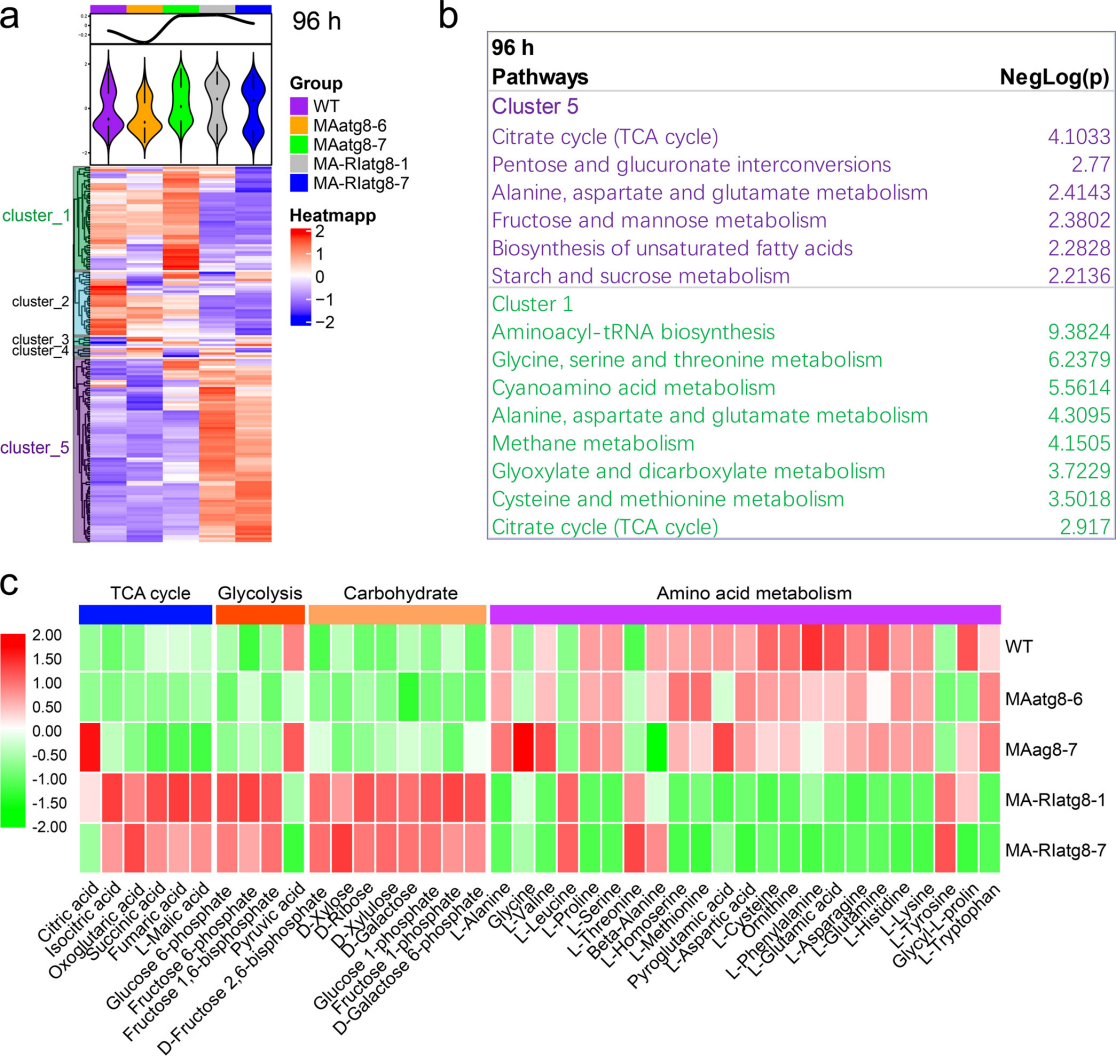
The metabolomics landscapes in WT and *MAatg8* overexpression and interference strains at 96 h. (a) Hierarchical clustering analyses of 96 h metabolomics data. Five modules were revealed by clustering analysis, the metabolites divided into the two major modules were used to perform pathway enrichment analysis, and the major pathways they govern are shown (b). (c) Differences in metabolites involved in the major pathways between WT and MAatg8 overexpression and interference strains at 96 h.

To quantitatively dissect the effects of *MAatg8* on cell metabolism, we compared the relative abundance of metabolites between WT and *MAatg8* overexpression and knockdown strains at 36, 96, and 168 h and clustered the change trends into five patterns (Fig. 3a, Fig. S6, supplemental file 3). From the heatmap and violin plot, we found that WT and *MAatg8* overexpression had similar metabolic landscapes but that they were significantly different from those of the knockdown strains. Most of the metabolites detected in this study were divided into two opposing change trends (upregulation and downregulation). We collected the metabolites in these two opposing trends, and the metabolites were used to perform pathway enrichment analysis (Fig. 3b and Fig. S6, supporting information 3). From the pathway enrichment analysis results, we observed that *MAatg8* interference increased the levels of metabolites involved in the tricarboxylic acid (TCA) cycle, pentose and glucuronate interconversions, alanine, aspartate, and glutamate metabolism, fructose and mannose metabolism, biosynthesis of unsaturated fatty acids, and starch and sucrose metabolism, while it decreased the levels of metabolites involved in aminoacyl-tRNA biosynthesis glycine, serine, and threonine metabolism, cyanoamino acid metabolism, alanine, aspartate, and glutamate metabolism, methane metabolism, and glyoxylate and dicarboxylate metabolism. We compared the metabolites involved in the TCA cycle, glycolysis, carbohydrate metabolism, and amino acid metabolism and found that *MAatg8* knockdown increased the metabolites involved in the TCA cycle and glycolysis and carbohydrate metabolism but the levels of free amino acids in WT and *MAatg8* overexpressing strains were significantly higher than those in *MAatg8* knockdown strains (Fig. 3c).

### Autophagy promotes membrane lipid turnover to ARA-rich TAGs.

**FIG 4:**
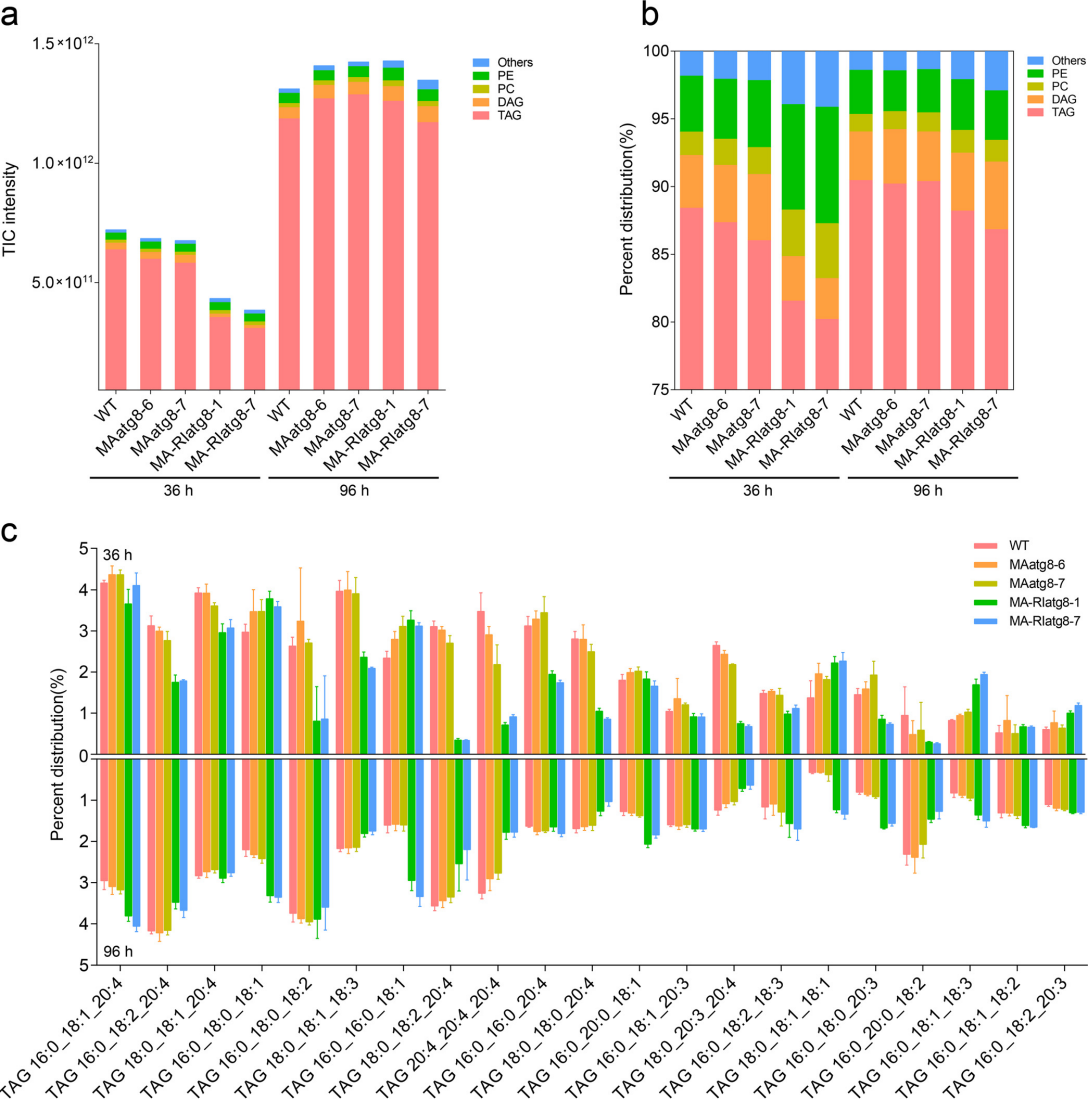
Lipidomics profiles detected in *M. alpina* from WT, *MAatg8* overexpression, and interference strains at 36 and 96 h. (a) Total ion chromatogram (TIC) intensity. (b) The distribution of major lipid classes. (c) The distribution of triglyceride profiles. TAG, triglyceride; DAG, diglyceride; PC, phosphatidylcholine; PE, phosphatidylethanolamine.

Next, lipidomic profiles of WT, *MAatg8*, and *MA-RIatg8* strains were analyzed at 36 and 96 h (supplemental file 2). Figure 4a shows that knockdown of *MAatg8* significantly decreased the total lipids (total ion chromatogram [TIC] intensity) in *M. alpina* at 36 h when the nitrogen sources were available, while the total lipid levels between WT, *MA-atg8*, and *MA-RIatg8* were similar at 96 h when the nitrogen sources were exhausted. Autophagy-related *MAatg8* had a significant effect on the total lipid synthesis of *M. alpina* during the growth phase, while this effect was obviously weakened after the nitrogen source was exhausted. Figure 4a and b shows that the major component in *M. alpina* was TAGs; however, the proportions of lipid classes among WT, overexpression, and knockdown strains were significantly different, even at 96 h, when the TIC intensities were similar. The *MA-RIatg8* strains had a higher proportion of phosphatidylcholine (PC) and PE, while a higher TAG proportion appeared to have a higher TFA accumulation. We further analyzed the fatty acid species compositions in the TAGs, and approximately 400 TAGs were synthesized in *M. alpina*. The prominent TAG species (accounting for more than 40% of the total TAG content) in *M. alpina* are shown in Fig. 4c. We found that *MAatg8* knockdown had an altered fatty acid composition at both 36 and 96 h. TAGs incorporated with arachidonic acid (ARA) (C_20:4_), such as TAG (20:4/20:4/20:4), TAG (18:0/18:2/20:4), and TAG (18:0/20:3/20:4), had a higher proportion in the WT and *MAatg8* overexpression strains, while TAGs incorporated with C_16_-C_18_ series fatty acids, such as TAG (16:0/16:0/18:1), TAG (18:0/18:1/18:1), TAG (16:0/18:0/20:3), and TAG (16:0/18:1/18:3), were higher in *MAatg8* knockdown strains. C_20:4_ synthesis appears to depend on the autophagy-related *MAatg8*. Target fatty acid analysis showed that, compared with that in WT strains, the proportion of ARA was 2.8 to 4.19% lower in *MAatg8* overexpression strains but 11.86 to 12.02% lower in *MAatg8* knockdown strains at 96 h. However, the proportion of C_18:1_ exhibited the opposite results (Table S4). We also analyzed the proportion of fatty acids in strains treated with ethanolamine. Surprisingly, compared with the control group, the external supply of ethanolamine significantly increased the proportion of ARA in WT and *MAatg8* overexpression strains, while its effects on *MAatg8* interference strains were limited (Table S4, supplemental file 3). This means that external supply of ethanolamine that increased ARA biosynthesis was dependent on the autophagy-related gene *MAatg8*.

## DISCUSSION

Autophagy is a cellular catabolic process in which various cytosolic components, such as mitochondria and proteins, are degraded and activated in response to nitrogen stress (23). Therefore, under nitrogen-limiting conditions, the *MAatg8* overexpression strains and WT *M. alpina* exhibited similar metabolomics profiles, while knockdown of *MAatg8* exhibited obvious differences in the metabolomics landscape.

The relationship between cell autophagy and lipid metabolism has been investigated for several decades, and the regulation of autophagy in lipid biosynthesis and degradation has been shown to differ among various cells and species (8–10, 24, 25). A commonly understood theory is that for lipid as the carbon and energy source, under nutrient limitation conditions, cell autophagy would be active and regulate lipid degradation to maintain cell energy supply and homeostasis (9, 26). However, this theory dose not explain the results obtained in this study. This study indicated the positive effects of autophagy on TFA synthesis under nitrogen-limiting conditions. In fact, cell autophagy promoting lipid accumulation has also been reported in specific cells or conditions. Studies regarding the effects of autophagy on adipocyte LDs demonstrated that autophagy regulates adipocyte LD formation through it effect on differentiation (11). Fewer LDs have been observed in the conidia of *MGatg1Δ* and *FGatg15Δ* of the plant pathogens Magnaporthe grisea and Fusarium graminearum, respectively (12, 27). In *Arabidopsis*, basal autophagy contributes to TAG synthesis, whereas inducible autophagy contributes to LD degradation (8). In *Chlamydomonas*, autophagy is required for the synthesis of TAG and recycling of ribosomal proteins under nutrient limitation conditions (28). In addition, PE-lipidated Atg8 has also been shown to localize to LDs and contribute to their formation (29, 30). In this study, the increase of TFA content in *MApsd2* overexpression strains and *MAatg8* overexpression strains cultured in supply of ethanolamine suggests that PE may act as a crucial molecular link between autophagy and lipid metabolism.

**FIG 5:**
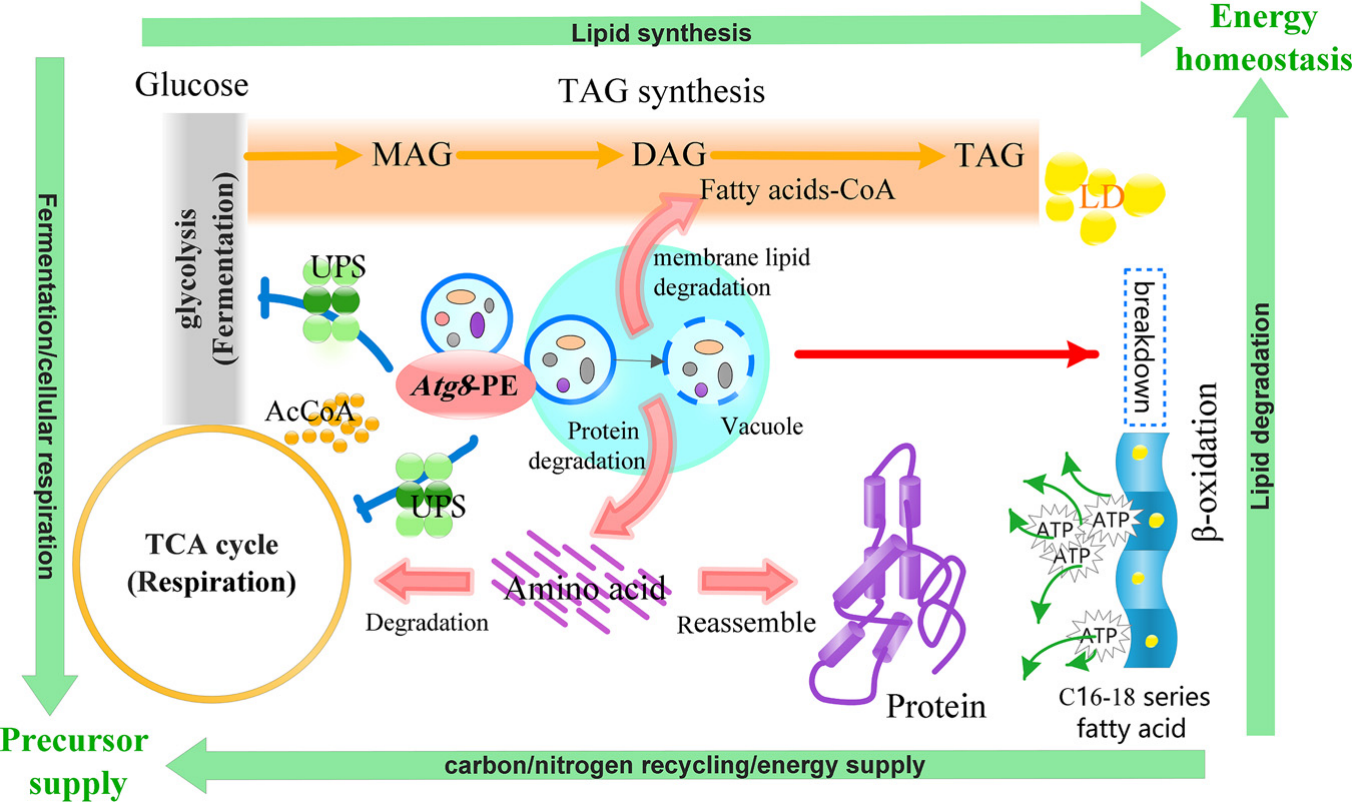
Schematic diagram showing the regulation role of cell autophagy on TAG biosynthesis. UPS, ubiquitin-proteasome system; TAG, triglyceride; DAG, diglyceride; MAG, monoacylglycerol; LD, lipid droplet; AcCoA, acetyl coenzyme A.

Compelling evidence suggests that nitrogen limitation-induced metabolic reprogramming plays an important role in lipid accumulation in oleaginous microorganisms (6). The comparison of metabolomics results and the biomass and total protein level between WT, *MAatg8*, and *MA-RIatg8* strains revealed that autophagy is involved mostly in the regulation of the nitrogen limitation-induced metabolic remodeling (Fig. 5). The *MAatg8* knockdown strains exhibited higher intensity of glycolysis, TCA cycle, and carbohydrate, indicating that downregulation of cell autophagy enhanced the oxidation of the carbon source and cell respiration under nitrogen limitation conditions, which was consistent with the higher biomass and glucose consumption rate in *MAatg8* knockdown strains. In contrast, autophagy activation enhanced protein degradation. The investigation by Jiao et al. demonstrated that glycolytic activity is negatively correlated with the autophagy level in liver cancer cells. The autophagic degradation of hexokinase 2 (HK2) was found to be involved in the regulation of glycolysis by autophagy. Specifically, the Lys63-linked ubiquitination of HK2 catalyzed by the E3 ligase TRAF6 was critical for the subsequent recognition of HK2 by the autophagy receptor protein SQSTM1/p62 for selective autophagic degradation (31). This means that the autophagy regulation of cellular respiration and fermentation balance was most likely mediated through the ubiquitin-proteasome system, which selectively degrades key proteins in the relevant pathways (Fig. 5). Biochemical and comprehensive proteomic analyses demonstrated that the abundance of intracellular proteins decreased in cells subjected to nitrogen limitation, and the products of protein degradation were a major source of the carbon skeleton for fatty acid synthesis (32–35). On the one hand, in *MAatg8* overexpression strains, the amino acids produced from protein degradation supplied free amino acid for protein reassembly through the upregulated aminoacyl-tRNA biosynthesis, and the remodeled intracellular proteomics landscape was beneficial for lipid synthesis (Fig. 5). On the other hand, the carbon skeleton released from free amino acid degradation would enter the TCA cycle and fatty acid synthesis pathways (Fig. 5).

In the present study, the lipidomic analysis found that autophagy-related *MAatg8* significantly regulated the lipid turnover. Studies using animal, yeast, and plant systems have established that autophagy functions in both breakdown and biogenesis of LDs (24, 25, 36). Under nitrogen stress conditions, autophagy-mediated membrane lipid (PC and PE) degradation supplied intermediates of the TAG biosynthesis pathway and promoted TAG accumulation (37, 38). In plants, autophagy has been implicated in the degradation of mitochondria, peroxisomes, endoplasmic reticulum, and chloroplasts, and fatty acids released from the membranes of these and other organelles may be used for TAG synthesis (39). In addition, the autophagy function in lipid catabolism is well recognized. It has been shown that *Arabidopsis* mutants in the peroxisome β-oxidation of fatty acids have greatly reduced seed oil content, demonstrating that peroxisome β-oxidation-mediated lipid turnover is an essential component for efficient seed oil accumulation (40). We noticed that the WT and *MAatg8* overexpression strain contain higher proportions of C_20:4_- and C_20:4_-bound TAG molecules (such as C_20:4_/C_20:4_/C_20:4_) than the *MAatg8* knockdown strains. Therefore, autophagy functions in fatty acid degradation (such as C_20:4_), but autophagy-mediated fatty acid degradation might be required for the net synthesis of fatty acids (40, 41). As fatty acids, their derivatives, and the carbon skeleton and energy released from fatty acid degradation have important structural or regulatory roles, such a balance is critical for the maintenance of normal metabolic and cellular functions in response to nutrient stress (42). We speculate that autophagy-mediated fatty acid composition alteration might contribute to achieving an appropriate balance between the synthesis and degradation of lipids (Fig. 5).

In the present study, investigating the effects of the MAatg8-PE conjugation system on TAG synthesis raises the interesting conclusion that cell autophagy is required for the synthesis of ARA-rich TAG in oleaginous fungal *M. alpina* under nitrogen-limiting conditions. The regulation of cell autophagy through the MAatg8-PE conjugation system-related gene overexpression or exogenous supply of compounds would be an efficient strategy to increase and maintain biomass productivity when high lipid content is obtained under nitrogen starvation. Our metabolomics and lipidomics analyses strongly support the hypothesis that cell autophagy is essential for regulating the nitrogen limitation-induced metabolic alteration and metabolic flux remodeling, which were recognized as key factors for the supply of the carbon skeleton and energy required for fatty acid synthesis. Our findings help develop new strategies to achieve more biomass and maximum lipid productivity for oleaginous microorganisms.

## MATERIALS AND METHODS

### Strains and media.

Wild-type *M. alpina* ATCC 32222 was used as a control, the uracil-auxotrophic strain *M. alpina* MAU1 (CGMCC no. 8414) was used as a transformation recipient to construct the recombinant strain, Escherichia coli strain TOP 10 (kanamycin resistant) was used for plasmid construction and maintenance, Agrobacterium tumefaciens AGL-1 (rifampin resistant) was used as a mediator for transforming *M. alpina*, and the binary plasmid pBIG2-ura5s-ITs was used as a vector.

E. coli TOP10 and A. tumefaciens AGL-1 were cultured in LB and yeast extract peptone (YEP; 10 g/L yeast extract, 10 g/L tryptone, 5 g/L NaCl) media, respectively. The preparation methods of the media used for A. tumefaciens*-*mediated transformation, including antibiotic-free minimal medium (MM), induction medium (IM), and uracil-free synthetic-complete (SC) agar plates, have been described previously (43). *M. alpina* activation in GY medium and flask fermentation in nitrogen-limited Kendrick medium were performed as described previously (44).

### Plasmid construction.

MAatg8 (accession number: MT680816) and MApsd2 (accession number: MT840666) were amplified from *M. alpina* ATCC 32222 cDNA. The genes were double digested with HindIII and SmaI, followed by ligation into pBIG2-ura5s-ITs treated with the same restriction enzymes (Thermo Fisher, USA). The resulting MAatg8/MApsd2 expression plasmids were named pBIG2-ura5s-MAatg8 and pBIG2-ura5s-MApsd2 and transformed into A. tumefaciens AGL-1 by electroporation (2.5 kV, 5.0 ms). The RNA interference technology of *M. alpina* established by our group was used in this study as described previously (20). Hairpin RNAs were expressed using the binary vector pBIG2-ura5s-ITs. Target gene-specific sequences (5′–3′, GGCCAGTTCGTTTATGTCATCCGCAAGCGTATCAAGCTCTCACCCGAGAAGGCTATCTTTATCTTTGTCAATGAGGTCCTCCCTCCAACA, 90 bp) were synthesized and inserted into the multicloning sites located upstream and downstream of the internal transcribed spacer (ITs) sequence with forward and reverse orientations, respectively (General Biosystems, Anhui, China). The resulting MAatg8 RNA interference plasmid was named pBIG2-ura5s-RI-MAatg8 and electrotransformed into A. tumefaciens AGL-1 by electroporation (2.5 kV, 5.0 ms). The primers used in these steps are listed in Table S1.

### ATMT and validation of transformants.

A. tumefaciens-mediated transformation of *M. alpina* MAU1 was performed as described previously (43). A single bacterial colony of A. tumefaciens harboring the vector was successively activated in YEP liquid medium and MM. Subsequently, A. tumefaciens was diluted and cultured in induction medium (IM) containing acetosyringone. Next, A. tumefaciens was gradient-diluted to an appropriate concentration and cocultured with germinating spores (10^7^ spores/mL, *M. alpina* MAU1). Uracil-free synthetic-complete (SC) agar plates containing cefotaxime and spectinomycin were used to screen positive colonies. The DNA of transformants was extracted by using a biospin fungus genomic DNA (gDNA) extraction kit (Bioflux, China). The presence of transfer DNA (T-DNA) was identified through PCR, and the PCR product was collected and purified using a DNA purification kit (Thermo Scientific, USA) and sent for sequencing (BGI, China).

### Culture of *M. alpina* and sample collection.

In this study, strains including one *M. alpina* ATCC 32222 (WT), two *MAatg8* overexpression (*MAatg8*-6 and *MAatg8-7*), two *MAatg8* RNA interference (*MA-RIatg8-1* and *MA-RIatg8-7*), and three *MApsd2* (*Mapsd2-3*, *Mapsd2-5*, and *MApsd2-6*) overexpression strains were selected for further fermentation to analyze the effects of cell autophagy on cell growth, metabolism, and lipid biosynthesis. Mycelium and the supernatant were harvested at 36, 96, and 168 h and stored at −80°C for further use. For each strain and time point, three biological repeats were used.

### Determination of total proteins, biomass, residual glucose, and fatty acid profiles.

The harvested mycelia were stored at −80°C for more than 12 h and then freeze dried using a lyophilizer. Total protein was extracted using lysis buffer (8 M urea, 1% SDS, 0.1% phenylmethylsulfonyl fluoride [PMSF], 150 mM Tris-HCl [pH 8.0]) and quantified using a bicinchoninic acid (BCA) method. Biomass, residual glucose determination, total fatty acids extraction, and gas chromatography–mass spectrometry (GC-MS) analyses were performed as described previously (44).

### Analysis of cell autophagy based on transcriptomics and proteomics information.

Autophagy-related biological process and genes were identified from our previous genome-wide gene ontology (GO) annotation and enrichment results (19). The fold change of autophagy-related genes prior to and after nitrogen limitation was calculated using the transcriptomics and proteomics data sets that we reported previously (19, 20). On that basis, the fold change and correlation of these selected genes at mRNA and protein level were analyzed. In order to understand the biological process of the significantly changed genes involved in detail, a GO enrichment analysis was performed for upregulated and downregulated genes, respectively.

### Metabolomic and lipidomic analysis.

Sample preparation and metabolites/lipid extraction and identification were performed as described previously (44, 45). For metabolomic analysis, fresh mycelia were extracted using MeOH:water (1:1). Extracts were dried in a vacuum centrifuge, resuspended in MeOX-pyridine and *N*-methyl-*N*-(trimethylsilyl)trifluoroacetamide (MSTFA) with 1% trimethylsilyl chloride (TMCS) for derivatization, and analyzed by GC-MS (Thermo Fisher Scientific, Boston, MA, USA) equipped with an RTX-5MS column (30 m by 0.25 mm by 0.25 μm, Restek, Bellefonte, PA, USA). For lipidomic analysis, freeze-dried mycelia were extracted with methyl tert-butyl ether. The lipidomic analysis was conducted using a Dionex UltiMate 3000 UPLC system (Santa Clara, CA, USA) coupled to a HESI probe with a Q-Exactive Orbitrap mass spectrometer (Thermo Fisher, CA, USA). The “raw” format files were converted to “abf” format using the ABF converter. The MSDIAL4.20 equipped with the DB_FiehnBinbase-FiehnRI and LipidMsmsBinaryDB-VS46-FiehnO database was used for metabolite and lipid molecule identification, respectively (46). All annotations were manually checked again. The mass spectrometry data have been deposited to MetaboLights with the data set identifier MTBLS4103.

### RT-qPCR analysis.

Total RNA of *M. alpina* was extracted using TRIzol reagent (Invitrogen, Thermo Fisher Scientific, Inc.) and reverse-transcribed into cDNAs using a PrimeScript RT reagent kit with gDNA Eraser (TaKaRa, Dalian, China). Real-time quantitative PCR (RT-qPCR) analysis was performed using SYBR green qPCR master mix (TaKaRa) and a Bio-Rad CFX384 real-time PCR system (primers listed in Table S1, supplemental file 3). *M. alpina* 18S rRNA was used as an internal control.

### Statistical analysis.

All data are presented as means ± standard deviations representing at least three independent experiments. SPSS software (SPSS standard version 19.0, SPSS, Inc.), GraphPad Prism version 6 (Graph Pad Software, San Diego, CA, USA), and Adobe Illustrator CS5 (Adobe, San Jose, CA, USA) were used to perform the statistical analyses and visualizations. Paired two-tailed *t* tests were used for two-group comparisons, and one-way analysis of variance with Tukey’s test was used for multiple comparisons. Statistical significance was defined as *P < *0.05. Principal-component analysis (PCA) was performed using Simca 14.1 software. Hierarchical clustering analysis and pathway enrichment analyses were performed using the online software MetaboAnalyst 4.0 (http://www.metaboanalyst.ca/) (47). The ggplot2 package in R software was used to perform the statistical analyses and visualizations.

### Data availability.

The mass spectrometry of metabolomics and lipidomics data have been deposited to the MetaboLights with the data set identifier MTBLS4103.
