# Supplementary material for: Autophagy Improves ARA-Rich TAG Accumulation in Mortierella alpina by Regulating Resource Allocation
Source: Microbiol Spectr. 2022 Feb 9;10(1):e01300-21. doi: 10.1128/spectrum.01300-21 (PMC8881083; doi:10.1128/spectrum.01300-21)
Supplement: SUPPLEMENTAL FILE 3 — Supplemental material. Download SPECTRUM01300-21_Supp_3_seq11.pdf, PDF file, 0.8 MB [file spectrum01300-21_supp_3_seq11.pdf]

Supporting Information _ Tables and Figures

Autophagy Improves ARA-rich TAG Accumulation in *Mortierella alpina* by Regulating of Resource Allocation

Hengqian Lu[1,2], Haiqin Chen[1,2,3,4]*, Xin Tang[1,2], Qin Yang[1,2], Hao Zhang[1,2,4], Yong Q. Chen[1,2,3,5] ,Wei Chen[1,2,3]

[1] State Key Laboratory of Food Science and Technology, Jiangnan University, Wuxi, Jiangsu 214122, China

[2] School of Food Science and Technology, Jiangnan University, Wuxi, Jiangsu 214122, China.

[3] National Engineering Research Center for Functional Food, Jiangnan University, Wuxi, Jiangsu 214122, China

[4] (Yangzhou) Institute of Food Biotechnology, Jiangnan University, Yangzhou 225004, China

[5] Department of Cancer Biology, Wake Forest School of Medicine, Winston-Salem, North Carolina, United States of America

***Corresponding author :** Haiqin Chen

Phone: 0086-510-85197239; Fax: 0086-510-85197239;

E-mail: haiqinchen@jiangnan.edu.cn

Table S1. Primers used for genes amplification and reverse transcription-qPCR analysis

Primer name	Sequences (5'-3')	Genes information
Hispro F1	CACACACAAACCTCTCTCCCACT	Universal primers for the vector
TrpCR 1	CAAATGAACGTATCTTATCGAGATCC	pBIG2-ura5s-ITs
MAatg8-F	ATGGTCCGCTCAGCATTTCAA	Autophagy related atg8
MAatg8-R	TAGTCTGATTCCTCTGCGACTT	
RT-MAatg8-F	ATGTCATCCATCTACGAAGAG	Autophagy related atg8
RT-MAatg8-R	TCTGAGCCGAATGTGTTC	
MApsd2-F	ATGAACGACGCTTCATCAGCAG	Phosphatidylserine
MApsd2-R	TTACAGACGCACCCCGATG	decarboxylase2

Table S2. The effects of external supply of ethanolamine on biomass and total fatty acid level in *M.alpina*

	Control	EA treatment	Percent increase (%)
Biomass (g/L)			
WT	7.51±0.07	12.16±0.14	61.92
MAatg8-6	7.40±0.42	12.94±0.59	74.86
MAatg8-7	7.70±0.37	12.93±0.38	67.92
MA-RIatg8-1	9.92±0.60	13.44±0.11	35.48
MA-RIatg8-7	9.75±0.98	14.08±0.24	44.41
Total fatty acid (g/g MDW)			
WT	187.21±4.63	201.42±3.23	7.59
MAatg8-6	202.06±7.94	232.11±10.8	14.87
MAatg8-7	210.66±3.80	233.46±7.3	10.82
MA-RIatg8-1	172.35±3.19	172.37±10.7	0.01
MA-RIatg8-7	179.35±12.63	180.57±6.90	0.68

The EA (ethanolamine, 50 mM) were added in the culture medium at 48 h of fermentation, and the samples were collected at 96 h for total fatty acid analysis. None ethanolamine added samples were used as control.

Table S3. The effects of MAatg8 overexpression and ethanolamine supply on total fatty acid synthesis in *M. alpina*.

Strains	Total fatty acid content (g/L)		Percent increase (%)
	Control	EA supply	
WT	1405.95±0.32	2449.27±0.45	74.21
MA-atg8-6	1495.24±3.33	3003.50±6.37	100.87
MA-atg8-7	1622.08±1.41	3018.64±2.77	86.10
MA-RIatg8-1	1709.71±1.91	2316.65±1.18	35.5
MA-RIatg8-7	1748.66±12.38	2542.43±1.66	45.39

The ethanolamine (50 mM) were added in the culture medium at 48 h of fermentation, and the samples were harvested at 96 h for total fatty acid analysis. None ethanolamine added fermentation were used as control.

Table S4. Effects of MAatg8-PE conjugation on fatty acid profiles of *M. alpina*

Fatty acids		Content of fatty acids (% of TFA)				
		WT	MA-atg8-6	MA-atg8-7	MA-RIatg8-1	MA-RIatg8-7
Control	C14:0	1.44±0.04	1.5±0.040	1.53±0.02	1.91±0.09	1.93±0.11
	C16:0	15.42±0.79	16.86±0.60	18.04±1.10	19.03±1.13	20.06±0.83
	C18:0	11.49±0.11	11.62±0.30	11.54±0.46	10.68±0.74	9.05±0.97
	C18:1	11.96±1.19	12.87±0.65	13.43±0.81	23.32±1.51	23.43±1.24
	C18:2	15.57±0.24	15.92±0.41	15.7±0.32	11.84±0.35	12.19±0.28
	C18:3	4.34±0.08	4.21±0.06	4.09±0.12	4.60±0.11	4.93±0.17
	C20:3	2.70±0.11	2.71±0.03	2.76±0.07	3.40±0.020	3.35±0.23
	C20:4	37.09±2.11	34.29±1.40	32.92±0.97	25.23±1.79	25.07±1.32
EA supply	C14:0	0.97±0.02	0.78±0.01	0.94±0.01	1.84±0.08	1.73±0.12
	C16:0	15.96±0.48	14.7±0.24	16.24±0.29	21.66±0.49	20.94±0.09
	C18:0	11.11±0.15	11.35±0.15	11.51±0.01	9.81±0.57	10.07±0.12
	C18:1	11.3±0.49	7.13±0.18	11.23±0.06	27.15±0.81	24.98±0.65
	C18:2	6.98±0.16	7.6±0.16	8.70±0.3	6.34±0.59	8.16±0.86
	C18:3	5.00±0.08	5.08±0.03	4.97±0.14	5.00±0.13	4.98±0.12
	C20:3	4.94±0.01	4.58±0.05	4.81±0.03	5.35±0.28	6.00±0.12
	C20:4	43.73±0.97	48.77±0.8	41.61±0.71	22.85±0.35	23.14±0.69

EA, Ethanolamine treatment group, ethanolamine (50 mM) were added in the culture medium at 48 h of the fermentation. The samples were harvested at 96 h for fatty acids analysis.

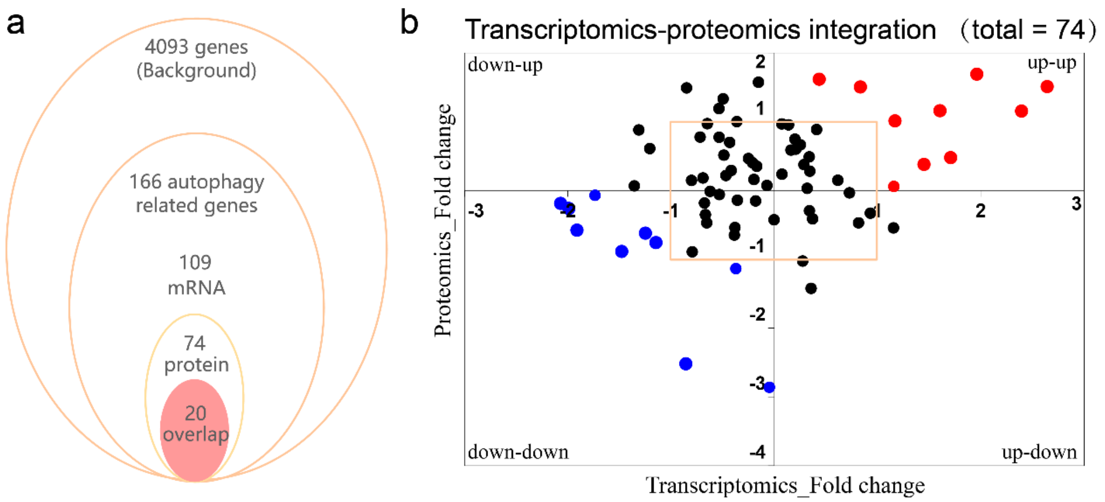

Figure S1. Cell autophagy in *M. alpina*. Based on our previous transcriptomics and proteomics datasets, cell autophagy-related genes and proteins were selected in this study. The change and correlation of these selected genes/proteins prior to and after nitrogen limitation were analyzed. **(a)** Number of annotated/identified genes and proteins involved in cell autophagy in *M. alpina*. **(b)** Correlation analysis of cell autophagy-related genes between mRNA and protein levels.

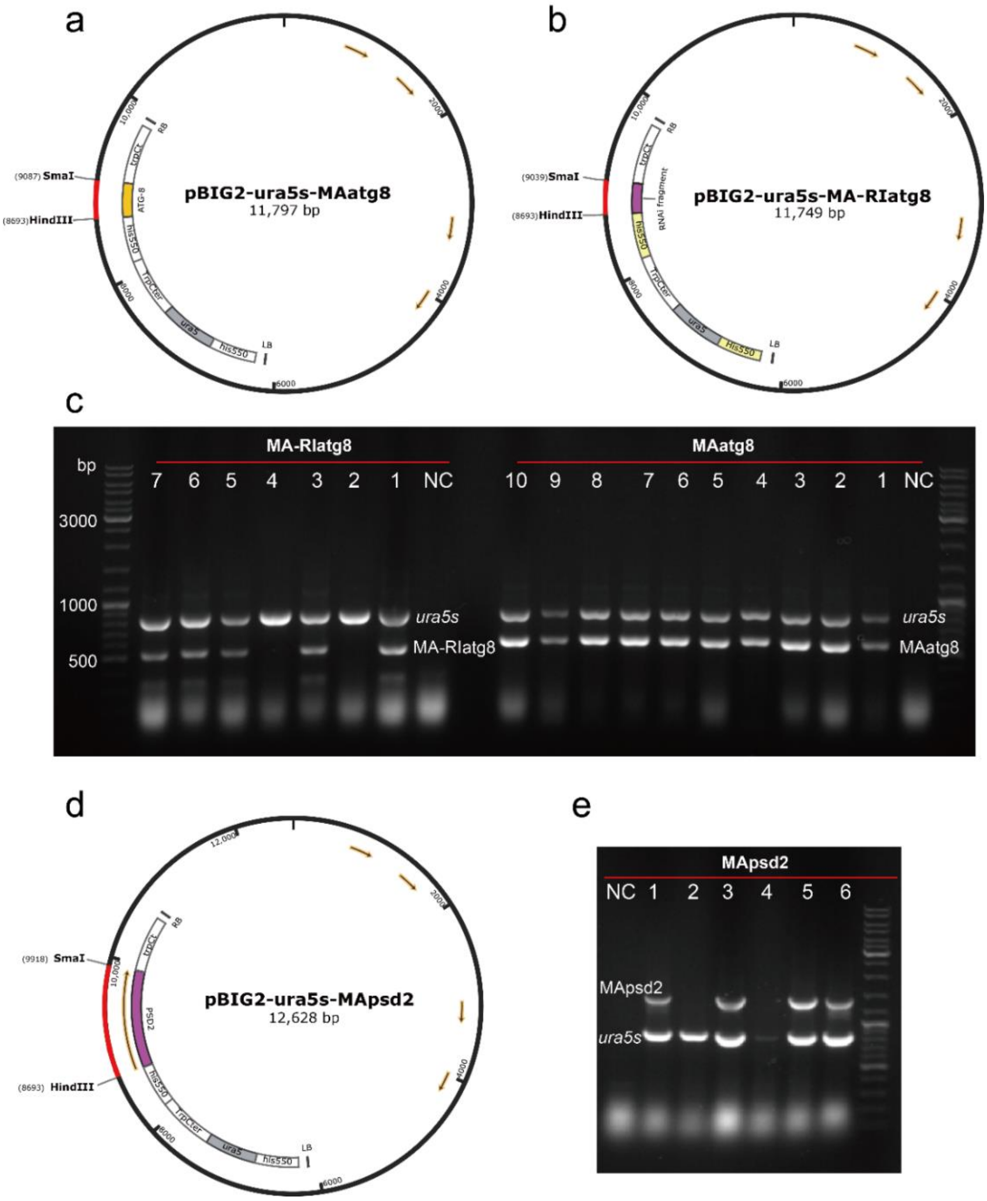

Figure S2. Construction of binary plasmids and PCR identification of MAatg8 recombinant strains. (a) Demonstration of construction of binary plasmids overexpression of MAatg8; **(b)** Demonstration of construction of binary plasmids RNA interference of MAatg8; (**c)** Electrophoresis results of the MAatg8 overexpression transformants and RNA interference transformants; (**d)** Demonstration of construction of binary plasmids overexpression of MApsd2; (**e)** Electrophoresis results of the MApsd2 overexpression transformants. NC, negative control. We obtained the coding sequence of MAatg8 through a basic local alignment search tool (blast). MAatg8 contains 378 base pairs and corresponds to 126 amino acids and only one atg8 coding sequence was found in *M. alpina* ATCC 32222. Using ATMT, the T-DNA region with MA-atg8 and Ma-RIatg8 and selection marker ura5s successfully inserted into the genome of the uracil auxotroph strain *M. alpina* MAU1. Ten overexpression and seven interference transformants were selected for identification, and the MA-atg8 (10/10) and MA-RIatg8 (5/7) segment were successfully amplified by PCR.

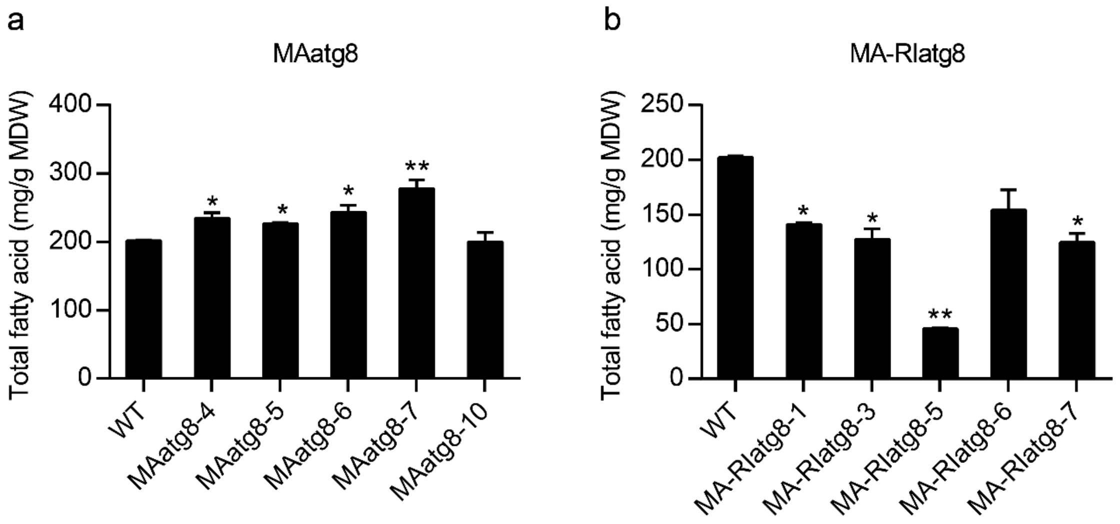

Figure S3. Fatty acid content of MAatg8 recombinant strains. (a) MAatg8 overexpression transformants; **(b)** MAatg8 RNA interference transformants. Five transformants for both overexpression and interference strains were selected to culture in Kendrick broth for 7 d. Total fatty acids (TFA) analysis results shown that TFA content was significantly increased and decreased in MA-atg8 and MA-RIatg8 strains, respectively. $p < 0.05$(*), $p < 0.01$(**). Based on the TFA content between the different transformants, WT, MAatg8-6/atg8-7, and MA-RIatg8-1/RI-atg8-7 strains were selected for further investigation of the relationship between cell autophagy and lipid metabolism in *M. alpina*.

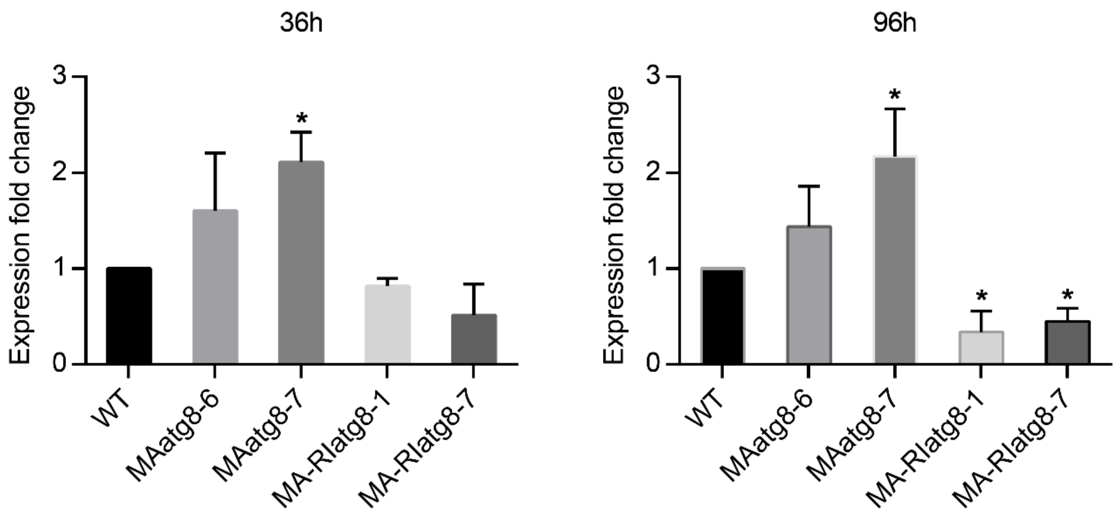

Figure S4. Analysis the changing fold of MAatg8 transcription levels at 36 h and 96 h by RT-qPCR. RT-qPCR analysis indicated that, compared with the WT strain, the expression level of MAatg8 was upregulated and downregulated in the overexpression and interference strains, respectively.

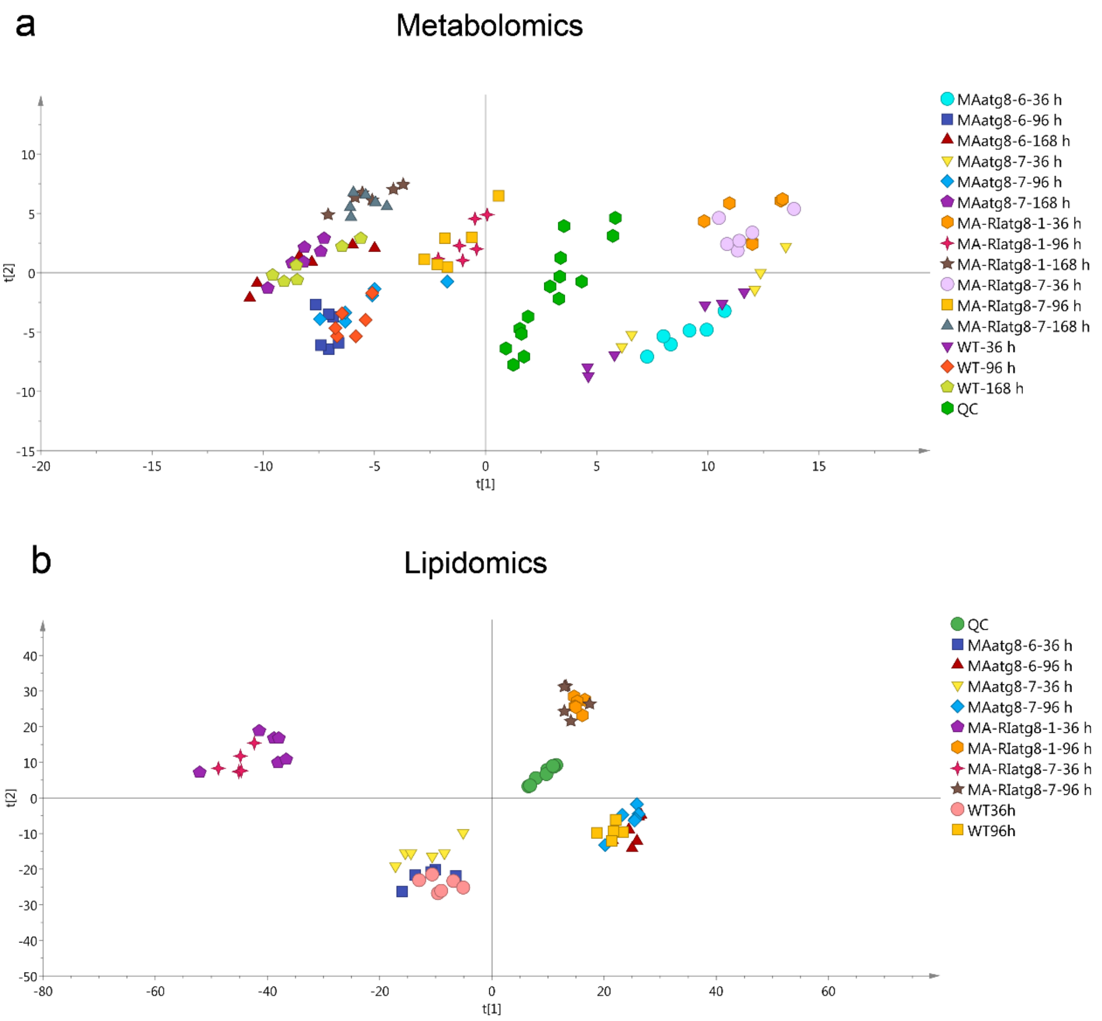

Figure S5. PCA polts of different time point samples for corresponding metabolomics (a) and lipidomics (b) datasets. Metabolomics datasets included 36 h, 96 h and 168 h samples; lipidomics dataset included 36 h and 96 h samples. For each time point, 5-6 repeats were proceed.

a

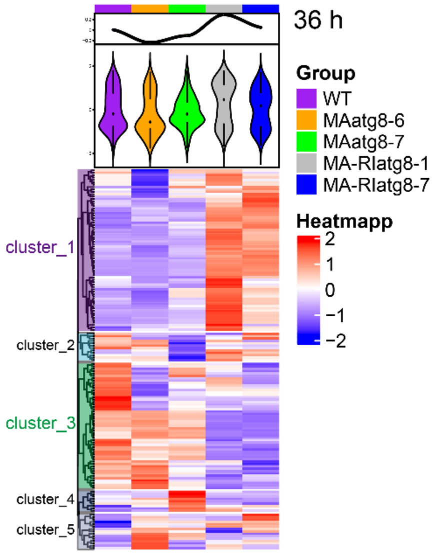

36 h Pathways	NegLog(p)
Cluster 1	
Citrate cycle (TCA cycle)	4.5431
Biosynthesis of unsaturated fatty acids	3.9357
Glyoxylate and dicarboxylate metabolism	3.4323
Glycine, serine and threonine metabolism	2.6451
Fructose and mannose metabolism	2.6344
Amino sugar and nucleotide sugar metabolism	2.4625
Cluster 3	
Aminoacyl-tRNA biosynthesis	13.822
Arginine biosynthesis	6.4413
Cyanoamino acid metabolism	5.0846
Lysine biosynthesis	4.9008
Alanine, aspartate and glutamate metabolism	3.7409
Glycine, serine and threonine metabolism	2.5301
Ubiquinone and other terpenoid-quinone biosynthesis	2.2863
Pentose and glucuronate interconversions	2.0717

b

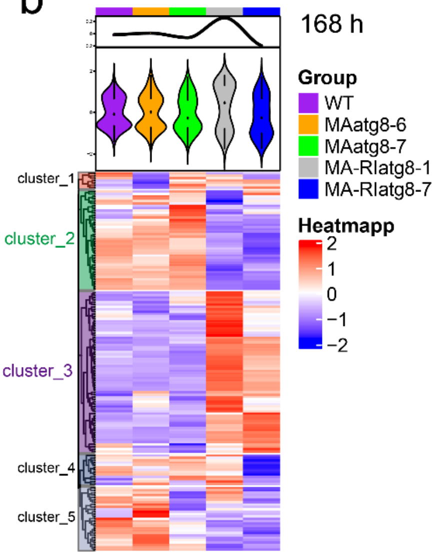

168 h Pathways	NegLog(p)
Cluster 3	
Citrate cycle (TCA cycle)	4.4765
Fructose and mannose metabolism	4.2313
Glyoxylate and dicarboxylate metabolism	3.3714
Alanine, aspartate and glutamate metabolism	2.6812
Butanoate metabolism	2.5959
Biosynthesis of unsaturated fatty acids	2.5445
Cluster 2	
Aminoacyl-tRNA biosynthesis	9.3824
Glycine, serine and threonine metabolism	8.1661
Cyanoamino acid metabolism	5.5614
Pentose and glucuronate interconversions	4.3168
Starch and sucrose metabolism	3.6825
Cysteine and methionine metabolism	3.5018
Alanine, aspartate and glutamate metabolism	2.6771
Methane metabolism	2.5676

Figure S6. The metabolomics landscapes in WT and MAatg8 overexpression and interference strains at 36 and 168 h. Hierarchical clustering analyses of 36 h **(a)** and 168 h **(b)** metabolomics data. Five modules were revealed by clustering analysis, the metabolites divided into the two major modules were used to perform pathway enrichment analysis, and the major pathways they govern are shown.
